# Efficacy and safety of maintenance therapy with pamiparib versus placebo for advanced gastric cancer responding to first‐line platinum‐based chemotherapy: Phase 2 study results

**DOI:** 10.1002/cam4.5997

**Published:** 2023-06-01

**Authors:** Fortunato Ciardiello, Yung‐Jue Bang, Andrés Cervantes, Mikhail Dvorkin, Charles D. Lopez, Jean‐Philippe Metges, Antonio Sánchez Ruiz, Mariona Calvo, Andrew H. Strickland, George Kannourakis, Kei Muro, Hisato Kawakami, Jia Wei, Christophe Borg, Zhaoyin Zhu, Neal Gupta, Robert J. Pelham, Lin Shen

**Affiliations:** ^1^ Dipartimento di Medicina di Precisione Università degli Studi della Campania Luigi Vanvitelli Caserta Italy; ^2^ Department of Internal Medicine Seoul National University College of Medicine Seoul South Korea; ^3^ Department of Medical Oncology, Biomedical Research Institute INCLIVA, CiberOnc University of Valencia Valencia Spain; ^4^ Algorithmic Biology Laboratory St. Petersburg Academic University, Russian Academy of Sciences St. Petersburg Russia; ^5^ Department of Medicine Knight Cancer Institute/Oregon Health and Science University Portland Oregon USA; ^6^ Institute of Oncology and Haematology CHU Morvan, Arpego Network Brest France; ^7^ Hospital Puerta de Hierro Majadohonda Madrid Spain; ^8^ Department of Medical Oncology ONCOBELL Program (IDIBELL), Institut Català d'Oncologia‐L'Hospitalet Barcelona Spain; ^9^ Department of Medical Oncology, Monash Health Monash University Melbourne Victoria Australia; ^10^ Ballarat Oncology & Haematology Services Wendouree Victoria Australia; ^11^ The Fiona Elsey Cancer Research Institute Ballarat Victoria Australia; ^12^ Department of Clinical Oncology Aichi Cancer Center Hospital Nagoya Japan; ^13^ Department of Medical Oncology Kindai University Faculty of Medicine Osaka‐Sayama Japan; ^14^ The Comprehensive Cancer Centre of Drum Tower Hospital Medical School of Nanjing University & Clinical Cancer Institute of Nanjing University Nanjing China; ^15^ University Hospital of Besançon Medical Oncology Department, CIC‐BT1431 Besançon France; ^16^ UMR1098, Molecular and Cellular Immune Therapies of Cancers INSERM Besançon France; ^17^ Clinical Development BeiGene Ltd Cambridge Massachusetts USA; ^18^ Department of Gastrointestinal Oncology, Key Laboratory of Carcinogenesis and Translational Research (Ministry of Education/Beijing) Peking University Cancer Hospital & Institute Beijing China

**Keywords:** clinical trial, gastric cancer, maintenance, phase 2, poly(ADP‐ribose) polymerase inhibitors

## Abstract

**Background:**

Poly (ADP‐ribose) polymerase (PARP) inhibitors (PARPi) are approved for the treatment of various solid tumors. In gastric cancer, genes commonly harbor mutations in the homologous recombination DNA repair pathway, potentially increasing sensitivity to PARPi. Pamiparib (BGB‐290) is a small molecule inhibitor of PARP1 and PARP2.

**Methods:**

The PARALLEL‐303 study (NCT03427814) investigated the efficacy and safety of pamiparib 60 mg orally (PO) twice daily (BID) versus placebo PO BID as maintenance therapy in patients with inoperable locally advanced or metastatic gastric cancer that responded to platinum‐based first‐line chemotherapy. The primary endpoint of this double‐blind, randomized, global phase 2 study was progression‐free survival (PFS) (RECIST version 1.1; per investigator assessment). Secondary endpoints included overall survival (OS) and safety.

**Results:**

In total, 136 patients were randomized 1:1 to receive pamiparib (*n* = 71) or placebo (*n* = 65). Median PFS was numerically longer with pamiparib versus placebo but did not reach statistical significance (3.7 months [95% confidence interval (CI): 1.9, 5.3] vs. 2.1 months [95% CI: 1.9, 3.8]; hazard ratio 0.8 [95% CI: 0.5, 1.2]; *p* = 0.1428). Median OS was 10.2 months (95% CI: 8.7, 16.3) in the pamiparib arm versus 12.0 months (95% CI: 8.2, not estimable) in the placebo arm. Overall, 8 patients (11.3%) in the pamiparib arm and 2 patients (3.1%) in the placebo arm experienced ≥1 TEAE leading to treatment discontinuation.

**Conclusions:**

Maintenance pamiparib did not meet statistical significance for superiority versus placebo for PFS, but was well tolerated with few treatment discontinuations; no unexpected safety signals were identified.

## 1 INTRODUCTION

Gastric cancer accounts for 5.6% of all diagnosed cancers and 7.7% of cancer deaths worldwide.^1^ Patients with newly diagnosed inoperable locally advanced or metastatic gastric cancer are typically treated with chemotherapy regimens containing a platinum‐based agent and a fluoropyrimidine, or with chemoradiation or best supportive care.^2^, ^3^ However, the duration of first‐line chemotherapy typically does not exceed 6 months, with limited survival benefit and significant toxicity associated with these regimens.^4^, ^5^, ^6^, ^7^, ^8^ Therefore, once maximum tumor reduction is achieved with first‐line chemotherapy, patients require a tolerable treatment option for maintenance therapy.

Small molecule inhibitors of poly (ADP‐ribose) polymerase (PARP) 1/2 interfere with DNA repair mechanisms.^9^, ^10^, ^11^ PARP protein inhibitors (PARPi) bind directly to, and inhibit the activity of, PARP enzymes, preventing DNA damage repair and trapping PARP‐DNA complexes at the DNA damage site.^11^, ^12^, ^13^, ^14^ The most commonly mutated genes in gastric cancer include members of the homologous recombination DNA repair pathway^15^, ^16^ (e.g., *breast cancer gene (BRCA)1* and *BRCA*2^10^, ^17^), resulting in tumors with homologous recombination deficiency (HRD).^9^ HRD leads to increased genomic instability and tumorigenesis,^9^ is characterized by loss of heterozygosity (LOH), and can increase tumor sensitivity to platinum‐based chemotherapy and treatment with PARPi.^18^, ^19^, ^20^ In tumors with HRD, both therapies have similar antitumor effects via the mechanism of synthetic lethality,^10^, ^11^ and HRD status has been shown to correlate with outcomes to PARPi.

PARPi are currently used in the treatment of various cancers, including as maintenance therapies.^16^, ^21^, ^22^, ^23^ PARPi‐based regimens are not yet recommended for use in patients with gastric cancer,^2^, ^3^ but are being actively investigated.^16^, ^24^ Notably, in a phase 3 study of patients with advanced gastric cancer progressing following first‐line therapy, overall survival (OS) was numerically improved with PARPi (olaparib) compared with placebo when used in combination with paclitaxel, supporting the potential clinical benefit of PARPi in gastric cancer, albeit the difference did not reach statistical sigificance.^25^ Numerous other studies of PARPi as monotherapy and in combination with targeted drugs and immune checkpoint inhibitors are ongoing in patients with gastric cancers.^16^, ^24^

Pamiparib is a small molecule PARP1/2 inhibitor currently approved in China for the treatment of patients with previously treated advanced ovarian cancer.^12^, ^26^ Pamiparib showed strong PARP‐trapping and anti‐proliferative activity, and brain penetration, in animal models with HRD tumors.^12^, ^27^, ^28^ In early‐phase clinical studies, single‐agent pamiparib demonstrated antitumor activity and was generally well tolerated in patients with advanced solid tumors and non‐mucinous high‐grade ovarian cancer.^27^, ^28^ Subsequent phase 2 studies in patients with previously treated BRCA1/2‐mutated ovarian cancer and HER2‐negative breast cancer have provided further evidence supporting the antitumor activity of pamiparib.^29^, ^30^ In addition, the combination of pamiparib with the anti‐PD‐1 antibody tislelizumab has been reported to be associated with clinical benefit in patients with advanced solid tumors in a phase 1 study.^31^ Here, we report the results of PARALLEL‐303, a phase 2 study designed to compare the efficacy, safety, and tolerability of maintenance therapy with pamiparib versus placebo in patients with inoperable locally advanced or metastatic gastric cancer that responded to first‐line platinum‐based chemotherapy.

## 2 MATERIALS AND METHODS

### 2.1 Study design

PARALLEL‐303 was a double‐blind, placebo‐controlled, randomized, multicenter, global phase 2 study (NCT03427814). This study changed from phase 3 to a phase 2 due to slow enrollment. Patients were recruited from 138 sites across 16 countries/regions (Australia, Belgium, China, Czech Republic, France, Georgia, Hong Kong, Hungary, Japan, Poland, Russia, Singapore, Spain, Taiwan, the United Kingdom, and the United States). All relevant Institutional Review Boards/Independent Ethics Committees approved this study, which was carried out in accordance with the International Conference on Harmonization Good Clinical Practice Guideline, the principles of the Declaration of Helsinki, and local laws and regulations. The full protocol (including eligibility criteria, endpoints, assessments, and statistical plan) is available in the Appendix S1.

### 2.2 Participants

Eligible patients were ≥18 years of age, with human epidermal growth factor receptor 2 (HER2)‐negative, histologically proven, inoperable locally advanced or metastatic adenocarcinoma of the stomach or gastroesophageal junction. All patients had an Eastern Cooperative Oncology Group performance status (ECOG PS) of ≤1, had received first‐line platinum chemotherapy for ≤28 weeks with a complete response (CR), as determined by the investigator per Response Evaluation Criteria in Solid Tumors version 1.1 (RECIST v1.1), or a confirmed partial response (PR) maintained for ≥4 weeks. Sufficient tumor tissue must have been provided for central laboratory determination of LOH status for randomization and exploratory biomarker analyses.

Exclusion criteria included prior treatment with a PARPi, anticancer therapy ≤14 days prior to randomization (including chemotherapy, biologic therapy, immunotherapy, investigational agents, anticancer Chinese medicine, or herbal remedies), diagnosis of myelodysplastic syndrome, and patients who received irradiation as part of prior first‐line treatment.

### 2.3 Interventions

Eligible patients were randomized 1:1 to receive pamiparib 60 mg orally (PO) twice a day (BID) or placebo 60 mg PO BID. Randomization was performed using an Interactive Response Technology system, took place ≤8 weeks after the last dose of first‐line platinum chemotherapy, and was stratified by region (China/Hong Kong/Taiwan vs. Australia/Europe/North America vs. Japan/South Korea vs. rest of world) and ECOG PS (0 vs. 1). There was no fixed duration of treatment in this study. Treatment with pamiparib or placebo continued until progressive disease (PD), unacceptable toxicity, death, or until another discontinuation criterion was met (including pregnancy, a major protocol deviation, withdrawal of consent, investigator's discretion, or start of a new anticancer therapy). A maximum of two dose reductions were permitted before the patient was permanently withdrawn from the study drug.

### 2.4 Endpoints

The primary endpoint of this study was progression‐free survival (PFS) by investigator assessment per RECIST v1.1. Following the screening tumor assessment, tumor imaging assessments were performed every 8 weeks (±7 days) after Day 1 (the first day of study drug administration). Secondary endpoints included OS, time to second subsequent treatment (TSST), objective response rate (ORR), duration of response (DoR), and time to response (TTR). ORR was defined as the proportion of patients with a best overall response of CR or PR per RECIST v1.1 by investigator assessment. Safety was evaluated through the incidence and severity of treatment‐emergent adverse events (TEAEs), including treatment‐related adverse events (TRAEs). TEAEs were graded according to National Cancer Institute‐Common Terminology Criteria for Adverse Events v4.03 or higher.

### 2.5 Statistical analyses

This study was designed to provide 80% power for assessment of the primary PFS endpoint using the following assumptions to determine the sample size: an overall type I error rate of 0.1 (one‐sided), a 1:1 randomization ratio, a median PFS for the placebo arm of 6.0 months, and a PFS hazard ratio (HR; pamiparib/placebo) of 0.63. A sample size of approximately 128 patients (64 per treatment arm) was required to achieve 85 PFS events within the planned study duration of approximately 26 months after the first patient was randomized to the study, assuming an estimated accrual period of 20 months.

The intent‐to‐treat analysis set included all randomized patients who were assigned to receive either pamiparib or placebo; used for PFS, OS, and TSST analyses. The efficacy evaluable analysis set included all randomized patients who had measurable disease at baseline and at least one post baseline tumor assessment (unless treatment was discontinued due to clinical progression or death); used for all efficacy analyses related to ORR, DoR, and TTR. The safety analysis set included all patients in the intent‐to‐treat analysis set who received any dose of pamiparib or placebo.

The final PFS analysis was performed when 85 PFS events had occurred. A stratified one‐sided log‐rank test at a 0.1 significance level incorporating the randomized stratification factors was used to compare treatment arms for the primary endpoint. The treatment effect on PFS was estimated by fitting a Cox proportional hazards model to the PFS, including treatment arm as a factor and stratification factors (region and ECOG PS) as strata. From this model, the HR was estimated and presented with a two‐sided 95% confidence interval (CI).

For the OS secondary endpoint, a stratified log‐rank test that incorporated the randomized stratification factors was used to compare treatment arms in the intent‐to‐treat analysis set. The HR and its two‐sided 95% CI were estimated using the stratified Cox proportional hazards model. Median OS, TSST, and DoR for each treatment arm was estimated using the Kaplan–Meier method, and the 95% CI was calculated using the Brookmeyer–Crowley method. The ORR and its exact two‐sided 95% CI were calculated for each treatment arm, with the Cochran–Mantel–Haenszel score test was used to compare treatment arms in the efficacy evaluable analysis set. TTR was assessed using descriptive statistics. Only patients with a response of CR or PR during the study were included in the DoR and TTR analyses.

## 3 RESULTS

### 3.1 Patients and treatment

**FIGURE 1:**
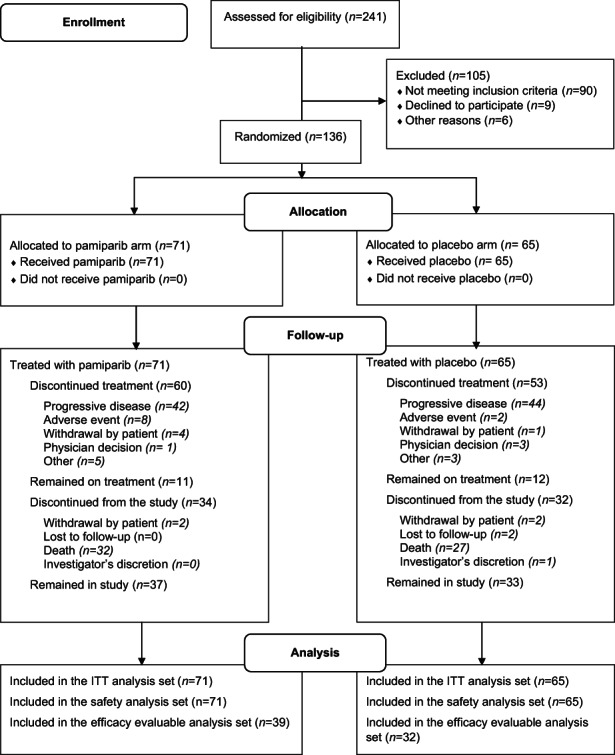
Patient flow. ITT, intent‐to‐treat.

Between July 23, 2018, and April 27, 2020, 136 patients were randomized to receive pamiparib (*n* = 71) or placebo (*n* = 65) (Figure 1). There were 136 patients in both the intent‐to‐treat and the safety analysis sets, and 71 patients in the efficacy evaluable analysis set. Patient demographics and baseline characteristics were generally balanced between treatment arms (Table 1). The median age of patients was 64 years, 66.9% of patients were male, and the majority of patients (54.5%) were White, followed by Asian (25.7%). At study entry, 55.1% of patients had an ECOG PS of 1 (80.9% had stage IV disease, 28.7% had undergone prior procedure(s) or surgery, and 5.9% had received prior radiotherapy; Table 1).

**TABLE 1:** Patient demographics and baseline characteristics.

	Pamiparib	Placebo
	(*n* = 71)	(*n* = 65)
Median age, years (range)	64.0 (39–82)	64.0 (27–85)
Age ≥65 years, *n* (%)	32 (45.1)	30 (46.2)
Sex, *n* (%)		
Female	25 (35.2)	20 (30.8)
Male	46 (64.8)	45 (69.2)
Race, *n* (%)		
Asian	20 (28.2)	15 (23.1)
Black or African American	0 (0.0)	2 (3.1)
Native Hawaiian or Other Pacific Islander	0 (0.0)	1 (1.5)
White	38 (53.5)	36 (55.4)
Other	1 (1.4)	3 (4.6)
Not reported/Unknown^a^	12 (16.9)	8 (12.3)
Geographic region, *n* (%)^b^		
China/Hong Kong/Taiwan	12 (16.9)	9 (13.8)
Australia/Europe/North America	52 (73.2)	51 (78.5)
Japan/South Korea^c^	6 (8.5)	5 (7.7)
Rest of world	1 (1.4)	0 (0.0)
ECOG PS, *n* (%)		
0	31 (43.7)	30 (46.2)
1	40 (56.3)	35 (53.8)
Number of prior regimens, *n* (%)		
1	66 (93.0)	60 (92.3)
2	3 (4.2)	5 (7.7)
≥3	2 (2.8)	0 (0.0)
Best overall response for last therapy, *n* (%)		
Complete response	4 (5.6)	6 (9.2)
Partial response	67 (94.4)	58 (89.2)
Stable disease	0 (0.0)	1 (1.5)
Solid tumor stage, *n* (%)^d^		
Stage IIA/B	3 (4.2)	1 (1.5)
Stage IIIA–C	4 (5.6)	6 (9.3)
Stage IV	59 (83.1)	51 (78.5)
Unknown	5 (7.0)	7 (10.8)
Prior anticancer therapy, *n* (%)		
Platinum‐based therapy	71 (100.0)	65 (100.0)
Radiotherapy	3 (4.2)	5 (7.7)
Procedures or surgery	25 (35.2)	14 (21.5)

Abbreviation: ECOG PS, Eastern Cooperative Oncology Group performance status.

^a^ “Not reported” and “Unknown” include patients from France who did not consent to the collection of race information.

^b^ Geographic regions are presented as per the groupings used as stratification factors during randomization.

^c^ No patients were enrolled in South Korea.

^d^ Solid tumor stage at screening.

At data cutoff (June 18, 2020), median follow‐up duration was 8.0 months (pamiparib arm, 7.9 months; placebo arm, 8.0 months). Seventy patients (51.5%) remained in the study, and 23 (16.9%) remained on treatment (Figure 1). Median duration of exposure to study treatment was 2.4 months in the pamiparib arm and 1.9 months in the placebo arm. After study treatment discontinuation, 27 patients (38.0%) versus 31 patients (47.7%) in the pamiparib and placebo arms, respectively, received subsequent treatment (Table S1).

### 3.2 Efficacy

**FIGURE 2:**
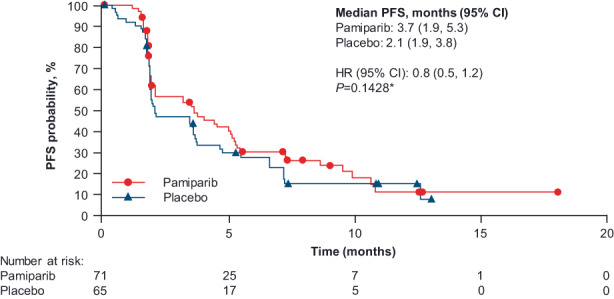
Kaplan–Meier plot of progression‐free survival per RECIST v1.1 in the intent‐to‐treat analysis set. PFS was estimated by the Kapan–Meier method, with 95% CIs estimated using the Brookmeyer–Crowley method. *p Value (one‐sided) was based on a stratified (region and ECOG PS) log‐rank test. CI, confidence interval; ECOG PS, Eastern Cooperative Oncology Group performance status; HR, hazard ratio; PFS, progression‐free survival; RECIST v1.1, Response Evaluation Criteria in Solid Tumors version 1.1.

In the intent‐to‐treat analysis set, median PFS was 3.7 months (95% CI: 1.9, 5.3) in the pamiparib arm and 2.1 months (95% CI: 1.9, 3.8) in the placebo arm (Figure 2), however, this difference was not statistically significant (HR 0.8; 95% CI: 0.5, 1.2; *p* = 0.1428). There were also no significant differences for the secondary efficacy endpoints between the pamiparib and the placebo arms (Table 2): Median OS was 10.2 (95% CI: 8.7, 16.3) versus 12.0 months (95% CI: 8.2, not estimable), and median TSST was 9.8 (95% CI: 8.1, 10.9) versus 9.7 months (95% CI: 7.5, 14.0), respectively.

**TABLE 2:** Summary of secondary efficacy endpoints.^a^

	Pamiparib	Placebo
	(*n* = 71)	(*n* = 65)
Median OS, months (95% CI)	10.2 (8.7, 16.3)	12.0 (8.2, NE)
Median TSST, months (95% CI)	9.8 (8.1, 10.9)	9.7 (7.5, 14.0)
	**(*n* = 39)**	**(*n* = 32)**
ORR, % (95% CI)	7.7 (1.6, 20.9)	6.3 (0.8, 20.8)
Median DoR, months (95% CI)	3.6 (3.5, NE)	NE (5.6, NE)
Median TTR, months (min, max)	3.7 (1.8, 7.3)	1.9 (1.9, 1.9)

*Note*: OS, TSST, and DoR were estimated by the Kapan–Meier method, with 95% CIs estimated using the Brookmeyer–Crowley method. ORR and its exact two‐sided 95% CI were calculated for each treatment arm, with the Cochran–Mantel–Haenszel score test was used to compare treatment arms. TTR was assessed using descriptive statistics.

Abbreviations: CI, confidence interval; DoR, duration of response; NE, not evaluable; ORR, objective response rate; OS, overall survival; TSST, time to second subsequent treatment; TTR, time to response.

^a^ OS and TSST were analyzed using the intent‐to‐treat analysis set; ORR, DoR, and TTR were analyzed using the efficacy evaluable analysis set.

In the efficacy evaluable analysis set, between the pamiparib and the placebo arms, respectively: ORR was 7.7% (95% CI: 1.6, 20.9) [3 PRs] versus 6.3% (95% CI: 0.8, 20.8) [2 PRs], the rate of stable disease was 46.2% (18 patients) versus 37.5% (12 patients), median DoR was 3.6 months and not evaluable, and median TTR was 3.7 months (range: 1.8, 7.3) versus 1.9 months (range: 1.9, 1.9).

### 3.3 Safety and tolerability

There was a similar incidence of TEAEs between treatment arms; 91.5% of patients receiving pamiparib and 93.8% of patients receiving placebo experienced ≥1 TEAE (Table 3). The most common TEAEs were anemia (36.6%), nausea (32.4%), and decreased appetite (26.8%) in the pamiparib arm, and abdominal pain (18.5%), nausea (16.9%), and asthenia (16.9%) in the placebo arm (Table 4). Overall, 40.8% of patients experienced at least one ≥Grade 3 TEAE in the pamiparib arm versus 30.8% in the placebo arm (Table 3).

**TABLE 3:** Summary of TEAE incidence (safety analysis set).

	Pamiparib	Placebo
	(*n* = 71)	(*n* = 65)
	*n* (%)	*n* (%)
Patients with ≥1 TEAE	65 (91.5)	61 (93.8)
Patients with ≥1 TRAE	55 (77.5)	34 (52.3)
≥Grade 3 TEAEs	29 (40.8)	20 (30.8)
≥Grade 3 TRAEs	19 (26.8)	6 (9.2)
Serious TEAEs	14 (19.7)	10 (15.4)
Serious TRAEs	1 (1.4)	3 (4.6)
TEAEs leading to dose modification	20 (28.2)	9 (13.8)
Leading to dose interruption	18 (25.4)	8 (12.3)
Leading to dose reduction	7 (9.9)	1 (1.5)
TRAEs leading to dose modification	14 (19.7)	7 (10.8)
Leading to dose interruption	12 (16.9)	6 (9.2)
Leading to dose reduction	7 (9.9)	1 (1.5)
TEAEs leading to treatment discontinuation	8 (11.3)	2 (3.1)
TRAEs leading to treatment discontinuation	5 (7.0)	1 (1.5)
TEAEs leading to death	2 (2.8)	2 (3.1)
TRAEs leading to death	0 (0.0)	1 (1.5)

Abbreviations: TEAE, treatment‐emergent adverse event; TRAE, treatment‐related adverse event.

**TABLE 4:** TEAEs reported in ≥10% of patients in either arm (safety analysis set).

	Pamiparib	Placebo
	(*n* = 71)	(*n* = 65)
	*n* (%)	*n* (%)
Patients with ≥1 TEAE	65 (91.5)	61 (93.8)
Anemia	26 (36.6)	8 (12.3)
Nausea	23 (32.4)	11 (16.9)
Decreased appetite	19 (26.8)	8 (12.3)
Asthenia	15 (21.1)	11 (16.9)
Diarrhea	13 (18.3)	7 (10.8)
Abdominal pain	8 (11.3)	12 (18.5)
Abdominal pain upper	12 (16.9)	7 (10.8)
Vomiting	17 (23.9)	1 (1.5)
Constipation	8 (11.3)	7 (10.8)
Aspartate aminotransferase increased	9 (12.7)	5 (7.7)
Alanine aminotransferase increased	8 (11.3)	5 (7.7)
Peripheral sensory neuropathy	4 (5.6)	9 (13.8)
White blood cell count decreased	8 (11.3)	3 (4.6)
Dysphagia	3 (4.2)	8 (12.3)
Weight decreased	8 (11.3)	1 (1.5)

Abbreviation: TEAE, treatment‐emergent adverse event.

The proportions of patients experiencing ≥1 TRAE were 77.5% and 52.3% in the pamiparib and placebo arms, respectively. The most common TRAEs were anemia (pamiparib 22.5%, placebo 10.8%), nausea (pamiparib 23.9%, placebo 6.2%), and decreased appetite (pamiparib 18.3%, placebo 1.5%).

Dose modifications due to a TEAE occurred in 28.2% and 13.8% of patients in the pamiparib and placebo arms, respectively. The most common TEAEs leading to dose modification were anemia (7.0%), vomiting (5.6%) and upper abdominal pain (4.2%) in the pamiparib arm, and anemia (3.1%) in the placebo arm.

Eight patients (11.3%) in the pamiparib arm and 2 patients (3.1%) in the placebo arm experienced ≥1 TEAE leading to treatment discontinuation (Table 3). The most common TEAEs leading to discontinuation were anemia (2.8%, 2 patients), nausea (1.4%, 1 patient), and increased alanine aminotransferase (1.4%, 1 patient) in the pamiparib arm, and nausea, abdominal pain, asthenia, and sepsis (1.5%, 1 patient each) in the placebo arm. Four patients experienced ≥1 TEAE that led to death: 2 patients (2.8%) in the pamiparib arm (cause listed as death under the general disorder and administration site conditions system organ class, and pneumonia; one patient each [neither were TRAEs]) and 2 patients (3.1%) in the placebo arm (due to sepsis [TRAE] and hepatic rupture [not TRAE], in 1 patient each).

## 4 DISCUSSION

In this phase 2 study, which investigated the efficacy and safety of maintenance pamiparib in patients with platinum‐sensitive locally advanced or metastatic gastric cancer, the median PFS observed in the pamiparib arm was numerically higher than that in the placebo arm (3.7 months vs. 2.1 months, respectively); however, this PFS improvement did not reach statistical significance, nor did the difference in OS between treatment groups.

The safety and tolerability profile of pamiparib in this study was consistent with that of pamiparib and PARPi in other tumor types and treatment settings.^27^, ^28^, ^29^, ^32^, ^33^ Maintenance therapy with pamiparib was tolerable and manageable in this patient population, with few treatment discontinuations due to TEAEs. In the pamiparib arm, the most common TEAEs were anemia, nausea, and decreased appetite, which is consistent with evidence for other PARPi.^25^, ^34^ These data indicate that there are no safety concerns with continued exploration of pamiparib in patients with gastric cancer.

Due to the difficulty in recruiting this patient population, the study was converted from a phase 3 to a phase 2 trial, and was therefore not sufficiently powered for OS analysis. Possible reasons for low recruitment to this study include changes in the treatment landscape for gastric cancer, such as additional first‐line, second‐line, and third‐line treatment options which were not recommended at the time of study design in 2018.^2^, ^3^, ^35^, ^36^ Therefore, the inclusion and exclusion criteria may have been outdated versus the current patient profile, in particular with regard to the exclusion of patients who were receiving ongoing therapy with nivolumab, which is now recommended in addition to platinum‐fluoropyrimidine doublet chemotherapy as standard therapy for selected patients.^2^ The maintenance setting design in this study requiring patients to have completed first‐line platinum‐based chemotherapy and demonstrated prior CR (or sustained PR for ≥4 weeks) may have limited the potential pool of eligible patients; patients who had received irradiation as part of prior first‐line treatment were also excluded. The inclusion of a placebo arm may also have contributed to low recruitment in Asian countries, where first‐line fluoropyrimidine continued after the cessation of platinum‐based chemotherapy.^37^ Therefore, the relatively small sample size restricting full assessment of the efficacy profile was a study limitation.

While some targeted agents have been approved for gastric cancer,^38^, ^39^, ^40^, ^41^, ^42^, ^43^, ^44^, ^45^ to date, no PARPi have been approved by the Food and Drug Administration in this setting. To our knowledge, this is the only randomized, double‐blind, placebo‐controlled phase 2 study that investigated the efficacy and safety of a PARPi in gastric cancer, in a global population. Maintenance pamiparib resulted in a numerically higher median PFS versus placebo in patients with inoperable locally advanced or metastatic gastric cancer responding to platinum‐based treatment, and was tolerable with a manageable safety profile in this patient population. Exploration of potential biomarkers of response or resistance to pamiparib maintenance therapy in this setting may help define patient populations for future investigations.

## AUTHOR CONTRIBUTIONS

**Fortunato Ciardiello:** Writing – original draft (equal); writing – review and editing (equal). **Yung‐Jue Bang:** Conceptualization (equal); investigation (equal); supervision (equal); writing – original draft (equal); writing – review and editing (equal). **Andrés Cervantes:** Investigation (equal); validation (equal); writing – original draft (equal); writing – review and editing (equal). **Mikhail Dvorkin:** Writing – original draft (equal); writing – review and editing (equal). **Charles D. Lopez:** Writing – original draft (equal); writing – review and editing (equal). **Jean‐Philippe Metges:** Writing – original draft (equal); writing – review and editing (equal). **Antonio Sánchez Ruiz:** Investigation (equal); validation (equal);Writing – original draft (equal); writing – review and editing (equal). **Mariona Calvo:** Writing – original draft (equal); writing – review and editing (equal). **Andrew H. Strickland:** Writing – original draft (equal); writing – review and editing (equal). **George Kannourakis:** Writing – original draft (equal); writing – review and editing (equal). **Kei Muro:** Investigation (equal); validation (equal); writing – original draft (equal); writing – review and editing (equal). **Hisato Kawakami:** Writing – original draft (equal); writing – review and editing (equal). **Jia Wei:** Writing – original draft (equal); writing – review and editing (equal). **Christophe Borg:** Writing – original draft (equal); writing – review and editing (equal). **Zhaoyin Zhu:** Data curation (equal); formal analysis (equal); methodology (equal); writing – original draft (equal); writing – review and editing (equal). **Neal Gupta:** Data curation (equal); writing – original draft (equal); writing – review and editing (equal). **Robert J. Pelham:** Conceptualization (equal); data curation (equal); formal analysis (equal); methodology (equal); supervision (equal); writing – original draft (equal); writing – review and editing (equal). **Lin Shen:** Conceptualization (equal); data curation (equal); formal analysis (equal); investigation (equal); methodology (equal); supervision (equal); writing – original draft (equal); writing – review and editing (equal).

## FUNDING INFORMATION

This study was sponsored by BeiGene, Ltd.

## CONFLICT OF INTEREST STATEMENT

AC received institutional research funding from Genentech, Merck Serono, Bristol Myers Squibb, MSD, Roche, BeiGene, Bayer, Servier, Lilly, Novartis, Takeda, Astellas, Natera, and Fibrogen; advisory board or speaker fees from Amgen, Merck Serono, and Roche. AHS, JW, MD, JPM, GK, and ASR have no conflicts of interest to disclose. CB received consulting or advisory fees from Roche, Sanofi, MSD, and Bayer. CDL received grants or has contracts with Taiho, Roche, AstraZeneca, Servier, and AACR. HK received grants or has contracts with Chugai Pharmaceutical Co. Ltd., Kobayashi Pharmaceutical CO. Ltd., Taiho Pharmaceutical, and Eisai Co. Ltd; consulting fees from Daiichi Sankyo; payment or honoraria from Bristol Myers Squibb, Eli Lilly Japan K.K, Ono Pharmaceutical, Daiichi Sankyo, Takeda Pharmaceutical Co. Ltd., Teijin Pharma Ltd, GlaxoSmithKline K.K, Bayer Yakuhin Ltd, MSD K.K., Chugai Pharmaceutical Co. Ltd, Merck Biopharma Co., Ltd, Yakult Pharmaceutical Industry, Taiho Pharmaceutical, and Otsuka Pharmaceutical Co., Ltd. KM declares institutional research funding from Astellas, Amgen, Solasia Pharma, Taiho, Merck Serono, Daiichi Sankyo, Parexel International, Pfizer, MSD, ONO Pharmaceutical, Sanofi, and Eisai; advisory board or speaker fees from AstraZeneca, ONO Pharmaceutical, Amgen, Chugai, Takeda, Taiho, Sanofi, Bristol Myers Squibb, Eli Lilly, and Bayer. LS declares consulting or advisory role with MSD, Bristol Myers Squibb, AstraZeneca, Daiichi Sankyo, Roche, Mingji Biopharmaceutical, Harbour BioMed, and Merck; speakers' bureau with Hutchison Whampoa and MSD; research funding from Nanjing Yaojieankang Biotechnology (Inst), Baiji Shenzhou (Beijing) Biotechnology Co. Ltd (Inst), Beijing Xiantong Biomedical Technology Co. Ltd (Inst), QiLu Pharmaceutical (Inst), and Zaiding Pharmaceutical (Inst). MC declares consulting and advisory role or speaker with Roche, Eisai, MSD, and Bayer; safety monitoring board with Nerviano. NG, ZZ, and RJP are employed by and have stock ownership in BeiGene. YJB declares institutional funding from Genentech/Roche, Merck Serano, Astellas, MSD, Daiichi Sankyo, and Amgen; consulting fees from MSD, Daiichi Sankyo, Samyang Biopharm, Hanmi, Merck Serano, Astellas, Alexo Oncology, Daewoong, and Amgen.

## ETHICS STATEMENT

All procedures followed were in accordance with the ethical standards of the responsible committee on human experimentation (institutional and national) and with the Helsinki Declaration of 1964 and later versions. Informed consent to be included in the study, or the equivalent, was obtained from all patients.

## Supporting information


Appendix S1

## Data Availability

On request, and subject to certain criteria, conditions, and exceptions, BeiGene, Ltd. will provide access to individual de‐identified participant data from BeiGene‐sponsored global interventional clinical studies conducted for medicines (1) for indications that have been approved or (2) in programs that have been terminated. BeiGene will also consider requests for the protocol, data dictionary, and statistical analysis plan. Data requests may be submitted to datadisclosure@beigene.com.
