# Supplementary material for: Efficacy and safety of maintenance therapy with pamiparib versus placebo for advanced gastric cancer responding to first‐line platinum‐based chemotherapy: Phase 2 study results
Source: Cancer Med. 2023 Jun 1;12(12):13145–54. doi: 10.1002/cam4.5997 (PMC10315793; doi:10.1002/cam4.5997)
Supplement: Supplementary file 1 — Appendix S1 [file CAM4-12-13145-s001.docx]

# **Supplementary Appendix for:**

Title: Efficacy and safety of maintenance therapy with pamiparib versus placebo for advanced gastric cancer responding to first-line platinum-based chemotherapy: Phase 2 study results

Fortunato Ciardiello, Yung-Jue Bang, Andres Cervantes, Mikhail Dvorkin, Charles D. Lopez, Jean-Philippe Metges, Antonio Sanchez Ruiz, Mariona Calvo, Andrew H. Strickland, George Kannourakis, Kei Muro, Hisato Kawakami, Jia Wei, Christopher Borg, Zhaoyin Zhu, Neal Gupta, Robert J. Pelham, Lin Shen

Corresponding author:

Lin Shen, MD, Professor, Chief of Department of GI Oncology, Peking University Cancer Hospital & Institute, 52 Fucheng Road, Haidian District, Beijing, China

Email: [linshenpku@163.com](mailto:linshenpku@163.com)

*Cancer Medicine*

**Supplement 1 (online only)**

**PARALLEL 303 Investigators List**

| **Country** | **Primary Investigator** | **Primary Center** |
| --- | --- | --- |
| Australia | Wong, Matthew | Central Coast Cancer Centre, Gosford Hospital |
| Australia | Strickland, Andrew | Monash Health |
| Australia | Chong, Geoff | The Northern Hospital |
| Australia | Kannourakis, George | Ballarat Oncology & Haematology Services |
| Australia | Dean, Andrew | St John of God Health Care |
| Belgium | Geboes, Karen | UZ Gent |
| Belgium | Loly, Catherine | CHU de Liège - Domaine Universitaire du Sart Tilman |
| Belgium | Rasschaert, Marika | UZ Antwerpen |
| Belgium | Vanderstraeten, Erik | AZ Maria Middelares - Campus Maria Middelares |
| Belgium | Bols, Alain | AZ Sint-Jan Brugge - Oostende - Campus Sint-Jan |
| Belgium | Van Custem, Eric | University Hospitals Leuven |
| China | Ma, Dong | Guangdong General Hospital |
| China | Yongqian, Shu | 1st Affiliated Hospital of Nanjing Medical U |
| China | Zhang, Xizhi | Northern Jiangsu people's hospital |
| China | Zhang, Longzhen | The Affiliated Hospital of Xuzhou Medical University East Hospital |
| China | Shen, Lin | Beijing Cancer Hospital |
| China | Bai, Yuxian | Harbin Medical University Cancer Hospital |
| China | Ying, Jieer | Zhejiang Cancer Hospital |
| China | Liao, Wangjun | Nanfang Hospital |
| China | Chen, Zhendong | The 2nd hospital of Anhui Medical U |
| China | Yin , Xianli | Hunan Cancer Hospital |
| China | Wei, Jia | Nanjing Drum Tower Hospital |
| China | Miao, Zhanhui | 1st Aff. Hospital of Xinxiang Medical U |
| China | Yuan, Ying | 2nd Aff Hosp of Zhejiang U Sch of Med |
| China | Gu, Kangsheng | First Affiliated Hospital Of Anhui Medical U |
| China | Pan, Yueyin | Anhui Provincial Hospital |
| China | Chen, Jia | Jiangsu Cancer Hospital |
| China | Tianshu, Liu | Zhongshan Hospital |
| China | Pan, Hongming | Sir RRS Hospital, Zhejiang University |
| China | Lv, Jing | The Affiliated Hospital of Qingdao U |
| China | Ba, Yi | Tianjin Cancer Hospital |
| China | Song, Min | 1st aff. hospital of Zhengzhou U |
| China | Xu, Ruihua | Sun Yat-sen University - Cancer Center |
| China | Deng, Yanhong | 6th Aff. Hospital, Sun Yat-Sen U |
| China | Chen, Lei | Cancer Hospital of Shantou University Medical College |
| China | Cai, Mingquan | 1st Aff. Hospital of Xiamen U |
| China | Zhang, Jingdong | Liaoning Cancer Hospital |
| China | Zhang, Jun | Rui Jin Hospital Shanghai Jiao Tong University School of Medicine |
| China | Niu, Zuoxing | Shandong Cancer Hospital |
| China | Zhuang, Zhixiang | The Second Affiliated Hospital of Soochow University |
| Czech Republic | Melichar, Bohuslav | Fakultni nemocnice Olomouc |
| Czech Republic | Zitnanska, Lucie | Thomayerova nemocnice |
| Czech Republic | Holeckova, Petra | Nemocnice Na Bulovce |
| France | Frin, Anne-Claire | Hôpital L'Archet 2 - CHU Nice |
| France | Tougeron, David | CHU Poitiers |
| France | Ducreux, Michel | Institut Gustave Roussy |
| France | Le Sourd, Samuel | Centre Eugene Marquis |
| France | Bachet, Jean-Baptiste | Hôpital de La Salpétrière |
| France | Vanbockstael, Julie | ICO Site Paul Papin |
| France | Borg, Christophe | CHRU Besançon - Hôpital Jean Minjoz |
| France | Metges, Jean-Philippe | Hôpital Morvan - CHRU de Brest |
| France | Samalin, Emmanuelle | Institut Régional du Cancer Montpellier (ICM) - Val d'Aurelle |
| France | Martin-Babau, Jerome | Hôpital Privé des Côtes d'Armor - Service oncologie |
| France | Artru, Pascal | Hopital Privé Jean Mermoz |
| France | Guimbaud, Rosine | Centre Hospitalier Universitaire (CHU) de Toulouse - Hopital |
| France | Vanbockstael, Julie | ICO Site René Gauducheau |
| Georgia | Dgebuadze, Eka | Tbilisi Cancer center LTD |
| Georgia | Melkadze, Tamar | LTD Acad.F.Todua Medical Center -Research Institute of Clinical Medicine |
| Georgia | Kortua, Tsira | Multiprofile Clinic Consilium Medulla LTD |
| Georgia | Matitashvili, Amiran | Scientific Research Center of Oncology LTD |
| Hong Kong SAR | Lam, Ka-On | Queen Mary Hospital |
| Hungary | Arkosy, Peter | Debreceni Egyetem Klinikai Központ |
| Hungary | Graf, Laszlo | Semmelweis Egyetem |
| Hungary | Dank, Magdolna | Semmelweis Egyetem |
| Hungary | Hitre, Erika | Országos Onkológiai Intézet |
| Hungary | Csoszi, Tibor | Jasz Nagykun Szolnok Megyei Hetenyi Geza Korhaz Rendeloint |
| Hungary | Babicz, Tamas | Szabolcs-Szatmar-Bereg Megyei Korhazak es Egyetemi Oktatokor |
| Hungary | Mangel, Laszlo | Pecsi Tudomanyegyetem Onkoterapias Intezet |
| Japan | Baba, Hideo | Kumamoto University Hospital |
| Japan | Hirao, Motohiro | National Hospital Organization - Osaka National Hospital |
| Japan | Kato, Ken | National Cancer Center Hospital |
| Japan | Kmatsu, Yoshito | Hokkaido University Hospital |
| Japan | Esaki, Taito | National Hospital Organization Kyushu Cancer Center |
| Japan | Yukisawa, Seigo | Tochigi Cancer Center |
| Japan | Tomita, Toshihiko | Hyogo College of Medicine College Hospital |
| Japan | Gamoh, Makio | Osaki Citizen Hospital |
| Japan | Satake, Hironaga | Kansai Medical University Hospital |
| Japan | Satoh, Taroh | Osaka University Hospital |
| Japan | Sugimoto, Naotoshi | Osaka International Cancer Institute |
| Japan | Kawakami, Hisato | Kindai University Hospital |
| Japan | Nakajima, Takako | St. Marianna University School of Medicine Hospital |
| Japan | Goto, Masahiro | Osaka Medical College Hospital |
| Japan | Tamura, Takao | Kindai University Nara Hospital |
| Japan | Muro, Kei | Aichi Cancer Center Hospital |
| Japan | Hirano, Gen | Japan Community Health Care Organization (JCHO) Kyushu Hospital |
| Japan | Otsu, Satoshi | Oita University Hospital |
| Japan | Hamaguchi, Tetsuya | Saitama Medical University International Medical Center |
| Japan | Shimada, Ken | Showa University Koto Toyosu Hospital |
| Poland | Sawka, Dariusz | Szpital Specjalistyczny w Brzozowie, Podkarpacki Osrodek Onkologiczny |
| Poland | Bryjak, Agnieszka | Salve Medica |
| Poland | Bar-Letkiewicz, Ilona | Clinical Research Center Sp. z o. o., Medic-R Sp. K. |
| Poland | Jankowski, Tomasz | Centrum Onkologii Ziemi Lubelskiej im. Sw. Jana z Dukli, Oddzial Onkologii Klinicznej z Pododdzialem |
| Poland | Wyrwicz, Lucjan | Centrum Onkologii - Instytut im. Marii Sklodowskiej-Curie, Klinika Onkologii i Radioterapii |
| Poland | Pikiel, Joanna | Szpitale Pomorskie sp. z o. o., Oddzial Onkologii i Radioterapii |
| Poland | Chudzik, Malgorzata | Mazowiecki Szpital Onkologiczny |
| Russia | Smolin, Alexey Vladimirovich | Main Military Hospital n.a. Burdenko |
| Russia | Akishina, Zinaida | GUZ Perm Regional Oncology Dispensary |
| Russia | Kovalenko, Nadezhda | Volgograd Regional Clinical Oncology Center |
| Russia | Makarova, Yulia | Guz Clinical Oncology Dispensary #1 |
| Russia | Topuzov, Eldar | GBOU VPO NorthWestern State Medical University n.a. I.I. Mec |
| Russia | Nechaeva, Marina | Arkhangelsk Regional Clinical Oncological Dispensary |
| Russia | Karachun, Alexey | N.N. Petrov Research Institute of Oncology |
| Russia | Lifirenko, Igor | GUZ Kursk Regional Oncology Dispensary |
| Russia | Tarasova, Anna | Samara Regional Clinical Oncology Dispensary |
| Russia | Dvorkin, Mikhail | BIH of Omsk Region "Clinical Oncology Dispensary" |
| Russia | Orlov , Sergey | The First Saint-Petersburg State Medical University named after Academican I.P. Pavlov |
| Singapore | Chang, Alex Yuang-Chi | Tan Tock Seng Hospital |
| Spain | Visa, Laura | Hospital del Mar |
| Spain | Pazo , Roberto | Hospital Universtiario Miguel Servet. |
| Spain | Sanchez Ruiz, Antonio | Hosp Universitario Puerta de Hierro Majadahonda |
| Spain | Zoilo, Reina | Hospital Universitario Virgen Macarena. |
| Spain | Ponz Sarvise, Mariano | Clinica Universidad de Navarra |
| Spain | Cervantes, Andres | Hospital Clínico Universitario de Valencia |
| Spain | Longo Munoz, Federico | Hospital Universitario Ramón y Cajal |
| Spain | Blanco, Montserrat | Hospital Universitario Gregorio Marañon |
| Spain | Camara, Juan Carlos | Hospital Universitario Fundacion Alcorcon |
| Spain | Cubillo, Antonio | Oncología, Centro Integral Oncológico Clara Campal. Servicio de Oncología, |
| Spain | Alsina, Maria | Hospital Universitario Vall dHebron |
| Spain | Martin, Marta | Hospital de La Santa Creu i Sant Pau |
| Spain | Calvo, Mariona | Institut Catala dOncologia |
| Taiwan | Huang, Chien-Tai | Chi Mei Medical Center |
| United Kingdom | Madhusudan , Srinivasan | Nottingham University Hospitals NHS Trust |
| United Kingdom | Arkenau, Hendrik-Tobias | Sarah Cannon |
| United Kingdom | Bhuva, Neel | East and North Hertfordshire NHS Trust |
| United Kingdom | Maisey, Nick | Guy's and St Thomas NHS Foundation Trust |
| United Kingdom | Hill, Esme | Royal Berkshire Hospital |
| United Kingdom | Madi, Ayman | The Clatterbridge Cancer Centre NHS Foundation Trust |
| United Kingdom | Ayers , Sarah | Peterborough And Stamford Hospitals |
| United States | Kio, Ebenezer | Goshen Center for Cancer Care |
| United States | Shehadeh, Nasfat | Novant Health & Presbyterian Healthcare - Presbyterian Hospital |
| United States | Dakhil, Shaker | Cancer Center of Kansas |
| United States | Lopez, Charles | Oregon Health and Science University |
| United States | Driscoll, Michael | Norton Cancer Institute |
| United States | Ucar, Antonio | Miami Cancer Institute |
| United States | Stanton, Thomas | St. Joseph Heritage Healthcare |
| United States | Gibson, Mike | Vanderbilt University Medical Center |
| United States | Gersten, Todd | Florida Cancer Specialists |
| United States | Crow, Mary | Renovatio Clinical |

**Complete study protocol**

BeiGene, Ltd. BGB-290-303 (Clinical study protocol) Protocol Amendment; 13 February 2020, Version 1.0

**Statistical Analysis Plan**

BeiGene, Ltd. BGB-290-303 (Statistical Analysis Plan); August 28, 2020, Version 1.0

**Full inclusion and exclusion criteria**

Inclusion criteria

Patients must have met all of the following criteria to be eligible for the study:

- Signed informed consent form
- Aged ≥18 years
- Histologically proven adenocarcinoma of the stomach or gastroesophageal junction, inoperable locally advanced or metastatic disease
  - Patients with gastric cancer overexpressing human epidermal growth factor receptor 2 (HER2) were not allowed
    - A negative result for HER2, as determined by local assessment, must have been documented in order for a patient to be eligible
  - Irradiation as part of prior first-line treatment was not allowed
- Availability of archival tumor tissue for central laboratory determination of homologous recombination deficiency status for randomization and exploratory biomarker analyses
  - Tumor tissue needed to originate from core or punch biopsy
  - Tumor tissue from fine-needle aspiration was not acceptable
- Received platinum-based first-line chemotherapy with a total of ≥8 platinum-containing 14-day cycles, ≥5 platinum-containing 21-day cycles, or ≥4 platinum-containing 28-day cycles for ≤28 weeks
- Confirmed partial response (PR) that was maintained for ≥4 weeks or complete response (CR) as determined by the investigator per Response Evaluation Criteria in Solid Tumors version 1.1 (RECIST v1.1)
- Ability to be randomized ≤8 weeks after last dose of platinum
- Eastern Cooperative Oncology Group performance status ≤1
- Ability to swallow whole capsules
- Ability to comply with study requirements and complete study questionnaires independently
- Adequate hematologic and end-organ function, as defined by the following laboratory results (obtained ≤14 days before randomization):
  - Absolute neutrophil count ≥1.5 × 10^9^/L
  - Platelet count ≥100 × 10^9^/L
  - Hemoglobin ≥9 g/dL (≥14 days after growth factor support or transfusion)
  - Estimated glomerular filtration rate ≥30 mL/min/1.73 m^2^ by the Modification of Diet in Renal Disease study equation ([www.mdrd.com](http://www.mdrd.com))
  - Total serum bilirubin ≤1.5 × upper limit of normal (ULN)
    - ≤4 x ULN, if Gilbert’s syndrome or if indirect bilirubin concentrations suggestive of extrahepatic source of elevation
  - Aspartate and alanine aminotransferase ≤3 × ULN
- Females of childbearing potential, nonsterile males, and female partners of nonsterile male study patients must agree to practice highly effective methods of birth control for the duration of the study and for at least 6 months after last study drug. Nonsterile males must avoid sperm donation for the duration of the study and for at least 6 months after last study drug

Exclusion criteria

Patients were excluded from the study for any of the following reasons:

- Unresolved acute effects of prior therapy of ≥Grade 2
  - Except for adverse events not considered a likely safety risk (e.g., alopecia, neuropathy, and specific laboratory abnormalities)
- Prior treatment with a poly (ADP-ribose) polymerase (PARP) inhibitor (PARPi)
  - Subtherapeutic exposure to a PARPi for ≤28 days was permissible provided it was not the most recent prior therapy
- Chemotherapy, biologic therapy, immunotherapy, investigational agent, anticancer Chinese medicine, or herbal remedies ≤14 days (or ≤5 half-lives, whichever was shorter) before randomization
  - Bisphosphonate and denosumab use was allowed on study, if administered at a stable dose >28 days before randomization
- Major surgical procedure, open biopsy, or significant traumatic injury ≤14 days before randomization, or anticipation of need for major surgical procedure during the course of the study
  - Placement of vascular access device is not considered major surgery
- Diagnosis of myelodysplastic syndrome
- Other diagnosis of malignancy
  - Except for surgically excised nonmelanoma skin cancer, adequately treated carcinoma in situ of the cervix, localized prostate cancer treated with curative intent, adequately treated low-stage bladder cancer, ductal carcinoma in situ treated surgically with curative intent, or a malignancy diagnosed >2 years ago with no current evidence of disease and no therapy 2 years before randomization
- Leptomeningeal disease or brain metastasis
- Active infection requiring systemic treatment, active viral hepatitis, or active tuberculosis
- Any of the following cardiovascular criteria:
  - Cardiac chest pain, defined as moderate pain that limits instrumental activities of daily living, ≤28 days before randomization
  - Symptomatic pulmonary embolism ≤28 days before randomization
  - Any history of acute myocardial infarction ≤6 months before randomization
  - Any history of heart failure meeting New York Heart Association Classification III or IV ≤6 months before randomization
  - Any event of ventricular arrhythmia Grade ≥2 in severity ≤6 months before randomization
  - Any history of cerebral vascular accident ≤6 months before randomization
- Previous complete gastric resection, chronic diarrhea, active inflammatory gastrointestinal disease, or any other disease causing malabsorption syndrome
  - Gastroesophageal reflux disease under treatment with proton pump inhibitors was allowed
- Active bleeding disorder, including gastrointestinal bleeding, as evidenced by hematemesis, significant hemoptysis, or melena ≤6 months before randomization
- Use ≤10 days (or ≤5 half-lives, whichever was shorter) before randomization or anticipated need for food or drugs known to be strong or moderate cytochrome P450 (CYP) 3A inhibitors or strong CYP3A inducers
- Pregnancy or nursing
  - Females of childbearing potential required a negative serum pregnancy test ≤7 days before randomization
- Significant intercurrent illness that may have resulted in the patient’s death before death from gastric cancer
- Known history of intolerance to the excipients of the pamiparib capsule

**Complete list of study endpoints**

Primary endpoints

- Progression-free survival, defined as the time from randomization to progressive disease per RECIST v1.1 by investigator assessment or death due to any cause, whichever occurred first

Secondary endpoints

*Efficacy assessment:*

- Overall survival, defined as the time from randomization to death due to any cause
- Time to second subsequent treatment, defined as the time from randomization until the second subsequent anticancer therapy or death after next-line therapy
- Objective response rate, defined as the proportion of patients with a best overall response of CR or PR per RECIST v1.1 by investigator assessment
- Duration of response, defined as the time from the first documented confirmed response of CR or PR to progressive disease per RECIST v1.1 by investigator assessment or death due to any cause, whichever occurred first
- Time to response, defined as the time from randomization to the first documented response of CR or PR per RECIST v1.1 by investigator assessment

*Safety assessment:*

- Incidence, timing, and severity of treatment-emergent adverse events, graded according to National Cancer Institute-Common Terminology Criteria for Adverse Events v4.03 or higher
- Safety and tolerability assessment of laboratory measurements, vital signs, and electrocardiogram findings

Exploratory endpoints

*Pharmacokinetics assessment:*

- Lowest observed plasma concentrations (C_trough_) at steady-state for patients who received pamiparib

*Patient-reported outcome assessments:*

- European Quality of Life 5-Dimensions 5-Levels Health Questionnaire
- European Organisation for Research and Treatment of Cancer Quality of Life Cancer Questionnaire (EORTC QLQ-C30)
- EORTC QLQ gastric cancer module (EORTC QLQ-STO22)

*Biomarker assessment associated with the pharmacodynamics, response, and resistance to pamiparib:*

- Including, but not limited to, expression and mutations of genes in the DNA damage response pathway, loss of heterozygosity, and relationship to efficacy and resistance to pamiparib

**Supplementary Table S1** Post-treatment subsequent anticancer therapies

|  | **Pamiparib**  **(*n*=71)** | **Placebo**  **(*n*=65)** |
| --- | --- | --- |
| Patients with any post-treatment anticancer therapy, *n* (%) | 27 (38.0) | 31 (47.7) |
|  |  |  |
| Subsequent anticancer systemic therapy used in ≥5% patients,  *n* (%)^a^ |  |  |
| Paclitaxel | 16 (59.3) | 10 (32.3) |
| Fluorouracil | 8 (29.6) | 14 (45.2) |
| Ramucirumab | 7 (25.9) | 9 (29.0) |
| Irinotecan | 5 (18.5) | 9 (29.0) |
| Oxaliplatin | 6 (22.2) | 7 (22.6) |
| Folinic acid | 5 (18.5) | 3 (9.7) |
| Radiotherapy | 4 (14.8) | 3 (9.7) |
| Paclitaxel and ramucirumab | 2 (7.4) | 4 (12.9) |

^a^Percentages are based on patients who received any post-treatment anticancer therapy.
